# Deviating HER2 status between primary breast carcinomas and their liver metastases

**DOI:** 10.3389/fonc.2025.1723716

**Published:** 2025-11-21

**Authors:** Yuexia Chen, Liu Yang, Wei Qu, Jianhong Tu, Suqing Cheng, Xingmei Guo

**Affiliations:** 1 Department of Pathology, Nanchang People’s Hospital (formerly The Third Hospital of Nanchang), Nanchang, Jiangxi, China; 2 Department of Pathology, Wuxi No.2 People’s Hospital, Wuxi, Jiangsu, China

**Keywords:** HER2 heterogeneity, HER2 low expression, prognosis, breast cancer primary lesion, breast cancer liver metastasis lesion

## Abstract

**Background:**

HER2 heterogeneity existed in metastatic breast cancer compared with primary breast cancer, and the differences may influence treatment decisions and prognosis for patients with metastatic breast cancer.

**Methods:**

To investigate HER2 heterogeneity in primary breast lesions and liver metastases and its impact on prognosis.HER2 expression in 87 patients with breast cancer liver metastases was categorized into three groups using IHC or FISH: HER2-0, HER2 low expression, and HER2-positive. The chi-square test was used to evaluate the association between HER2 heterogeneity and clinicopathological characteristics, while prognosis was analyzed using Kaplan–Meier survival and COX regression analyses.

**Results:**

HER2 heterogeneity was found in 38% of patients with primary breast cancer and liver metastases. Analysis of clinicopathological features showed that patients aged ≥ 50 years and those with high ER heterogeneity were more likely to display HER2 heterogeneity. Kaplan-Meier survival analysis showed no significant difference in OS or DFS between the HER2-low expression group and the HER2–0 groups, either in primary breast lesions or liver metastases. Similarly, no significant differences in OS or DFS were observed among patients aged ≥ 50 years. Notably, COX univariate and multivariate analyses identified heterogeneous PR expression as an independent risk factor for disease-free survival in this cohort of breast cancer patients.

**Conclusions:**

HER2 heterogeneity is present in both primary breast cancer and liver metastases. Ongoing monitoring of molecular markers, particularly HER2 expression, remains essential for breast cancer patients, as it may provide opportunities for therapeutic intervention.

## 1 Introduction

Breast cancer is among the most prevalent malignancies in women and remains one of the leading causes of cancer-related mortality worldwide (1). It is a highly heterogeneous disease that requires individualized therapeutic approaches based on distinct molecular characteristics. Key molecular markers guiding treatment include the expression of HER2, ER, and PR, gene mutations such as *BRCA1/2* and *PIK3CA*, and immune microenvironment indicators, including TIL and PD-L1 (2).

Human Epidermal Growth Factor Receptor 2 (*HER2*/erbB2) belongs to the Epidermal Growth Factor Receptor (EGFR) family. Overexpression of the HER2 protein promotes tumor growth and proliferation through MAPK/ERK and PI3K/AKT/mTOR pathways (3, 4). Numerous studies have shown that HER2 overexpression is associated with poor prognosis in several cancers, including breast cancer, and it has been identified as a major driver of tumorigenesis (5, 6). According to immunohistochemistry technology(IHC) to detect the expression level of HER2 protein, the HER2 expression status is divided into 0, 1+, 2+, 3+ Fluorescence *in situ* hybridization(FISH) technology was used to detect the amplification level of HER2 gene, and HER2 2+was further distinguished as HER2 negative and HER2 positive. HER2-targeted therapies have emerged as an essential strategy in cancer treatment, demonstrating remarkable efficacy in various cancers. Traditionally, anti-HER2 therapy has been considered effective only for patients with HER2-positive breast cancer, with limited benefit observed in HER2-negative cases (7). However, about 60% of HER2-negative breast cancers exhibit low HER2 expression (IHC 1+ or IHC 2+/FISH-). The DESTINY-Breast04 study provided strong evidence that trastuzumab delivers significant therapeutic benefit of trastuzumab compared with chemotherapy in advanced breast cancer with low HER2 expression, establishing HER2-low expression as a distinct therapeutic subtype of breast cancer (7, 8). HER2-low breast cancer is defined as tumors with HER2 protein low expression without detectable gene amplification, specifically characterized as immunohistochemistry (IHC) 1+ or IHC 2+ with fluorescence *in situ* hybridization (FISH) negative (9).In addition to HER2 positive and HER2 low expression, the DESTINY-Breast06 study also proposed the concept of HER2 ultra-low expression. HER2 ultra-low expression refers to IHC 0 patients with “≤ 10% invasive cancer accompanied by weak and incomplete cell membrane staining” in previous IHC 0. Therefore, HER2–0 expression was further subdivided: HER2–0 expression without cell membrane staining; HER2–0 expression is present in cell membrane staining, with the latter being HER2 ultra-low expression (10, 11). Despite the promising advances of HER2-targeted therapies in recent years for various cancers, their efficacy is often affected by drug resistance and the heterogeneity of HER2 expression (12).

There is increasing evidence that breast cancer evolves from its initial state as it progresses, exhibiting heterogeneity that includes not only changes in histological type but also variations in the expression of molecular markers (13). As early as 2001, Tanner et al. highlighted the heterogeneity of HER2 expression in metastatic breast cancer compared with primary breast cancer (14). Subsequent studies have confirmed differences in HER2 expression between primary breast cancer lesions and metastatic sites. These differences may affect treatment strategies and patient prognosis in metastatic breast cancer, carrying important clinical implications (15).

Although advances in therapeutics, such as cyclin-dependent kinase 4 and 6 (CDK4/6) inhibitors, immune checkpoint inhibitors, and novel anti-human epidermal growth factor receptor 2 (HER2) antibody-drug conjugates (ADCs)have improved treatment options, distant metastases remain a major cause of treatment failure and mortality in breast cancer. Common metastatic sites include the bone, lung, liver, and brain, with the liver being the most frequent site of metastasis for solid tumors and the third most common site for breast cancer (16–19). However, heterogeneity in molecular expression between liver metastases and primary breast cancer poses significant challenges in the treatment of advanced breast cancer.

As tumor progression involves dynamic biological changes, repeated HER2 evaluation during the course of breast cancer is necessary to improve access to effective anti-HER2 therapies. This study focuses on HER2 heterogeneity in liver metastases of breast cancer by analyzing HER2 expression in liver metastases and assessing heterogeneity patterns. Although many studies have investigated HER2 heterogeneity in primary breast cancer and metastases, most have focused mainly on differences between HER2-negative and HER2-positive states. Few have addressed HER2 low expression, and limited research has examined how HER2 heterogeneity influences tumor prognosis. So, we analyzed the relationship between HER2 heterogeneity(included HER2 low expression) in primary breast and liver metastatic lesions and the clinicopathological features and prognosis of breast cancer patients.

## 2 Materials and methods

### 2.1 Human tissue specimens

Between January 1999 and July 2019, a cohort of 87 female patients with liver metastases stemming from breast cancer was assembled at the Department of Pathology, The Third Hospital of Nanchang. Each participant had undergone surgical treatment for breast cancer followed by standard chemotherapy before the emergence of liver metastases. The female subjects in the study were aged between 25 and 78 years, with an average age calculated at 64 years. The majority of the pathological classifications were identified as non-specific invasive ductal carcinoma, comprising 76 instances. Additionally, the cohort included 3 cases of invasive lobular carcinoma, a single case of invasive neuroendocrine carcinoma, 4 instances of invasive micropapillary carcinoma, 1 case of medullary carcinoma, and 2 cases of mucinous carcinoma.

A detailed retrospective review was conducted on the tissue specimens collected from 87 patients diagnosed with invasive breast cancer. These patients had all undergone mastectomies at the Department of Pathology at The Third Hospital of Nanchang over a period spanning from January 1999 to July 2019. The criteria established for inclusion in this analysis were: 1. Pathological confirmation of invasive breast cancer for all subjects; 2. Receipt of breast cancer surgery and standard chemotherapy, subsequently developing liver metastases; 3. Acquisition of liver metastatic specimens via core needle biopsy; 4. The inclusion criteria mandated the availability of complete clinicopathological data, detailed postoperative pathology reports, and comprehensive follow-up information for each patient. For the consistency of the experiment, we performed HER2 IHC on all selected pathological sections of primary breast cancer and liver metastases from 1999 to 2019.

Exclusion criteria encompassed:1. Patients with bilateral breast cancer; 2. Individuals diagnosed with other malignant tumors; 3. Patients who succumbed to accidents (such as traffic incidents or other sudden illnesses) during the follow-up period.

The 87 breast cancer patients were categorized based on their HER2 status into HER2-0 (IHC 0), HER2 low expression (IHC 1+ or IHC 2+/FISH-), and HER2-positive (IHC 3+ or IHC 2+/FISH+) groups, as determined by IHC or fluorescence *in situ* hybridization (FISH) methods. Any variation in the expression levels among these three HER2 categories was defined as HER2 heterogeneous expression; otherwise, HER2 expression was deemed homogeneous. A five-year period from the date of surgery was established as the cutoff point for assessing five-year OS and DFS, which were evaluated through telephone follow-ups or imaging studies. All participants provided written informed consent.

### 2.2 Immunohistochemical test

Immunohistochemical staining was executed using the EnVision dual-phase protocol. Antibody kits for the ER designated as clone SP1 and for the PR designated as clone SP2 were procured from Fujian Maixin Company. The primary antibody targeting HER2, specifically the VENTANA anti-HER2/nue (4B5) Rabbit Monoclonal Primary antibody, was supplied by Roche. For the secondary detection, MaxVision-HRP formulated for both rat and rabbit antibodies was utilized, along with the chromogen solution DAB. Both the secondary antibody and the DAB chromogen were obtained from Fuzhou Mai Xin Co., Ltd.

IHC staining procedure: Tissue specimens were fixed in 10% neutral formalin such that tissue blocks were subsequently processed, dehydrated and embedded in dehydrated paraffin. Then these paraffin embedded tissues were carefully sectioned and cut extraordinarily thin, four micrometers in thickness. Deparaffinization and rehydration steps were carried out on the prepared sections: three separate washes with distilled water for 3 minutes each and finally three washes with PBS for 5 minutes each. Sections were treated with a pH 9.0 EDTA buffer followed by three minutes of high pressure steam, then allowed to return to ambient temperature to facilitate antigen exposure. The tissue samples were then subjected to further three washes with PBS, each of five minutes’ duration after these initial steps. The tissue was subsequently treated with a 3% hydrogen peroxide solution for 10 minutes in order to deactivate endogenous peroxidase enzymes. After this, three rinses with a PB were given to the sections, each for 5 minutes. After removing it, the samples were washed another trio of 5 min PBS washes. Immunohistochemical staining was performed (staining for ER, PR and HER2) meticulously, as per manufacturers’ protocol. In particular, thirty minutes were given for the HER2 primary antibody to be applied and incubated at room temperature upon the sections. After that, the cells were kept overnight at 4 °C with the primary antibody, after which were washed three times with PBS for five minutes each. Then the secondary antibody from Maixin Company was incubated at the room temperature for forty-five minutes. Furthermore, the colorimetric reaction was developed using the DAB (3,3-diaminobenzidine) chromogen purchased from Maixin Company and following PBS washes of 5 minutes for three times each. In the final steps the sections were counter stained with hematoxylin and permanently sealed after dehydration.

Result Interpretation:

The assessment of HER2 immunostaining was carried out in accordance with the 2024 Breast Cancer HER2 testing guidelines (20), employing the following classification criteria:

Category 0: Absence of HER2 staining or the presence of incomplete, barely perceptible membrane staining in 10% or fewer of the tumor cells.

Category 1+: Detection of incomplete and weak membrane staining in more than 10% of the tumor cell population.

Category 2+: Observation of intact membrane staining with weak to moderate intensity in over 10% of tumor cells, or strong membrane staining in up to 10% of tumor cells.

Category 3+: Presence of strong, complete, and uniform membrane staining in more than 10% of tumor cells.

(2) In the evaluation of IHC staining, a tumor sample was classified as positive for ER and PR if staining was present in the nuclei of at least one percent of the tumor cells, regardless of the staining intensity. This threshold indicates that a minimal proportion of tumor cells exhibit varying levels of hormone receptor expression. On the contrary, a tumor was considered negative for ER and PR when fewer than one percent of the tumor cell nuclei displayed any degree of staining or when no nuclear staining was observed at all (21).

### 2.3 Fluorescence *in situ* hybridization

The HER2 gene probe kit along with the protease enzyme were sourced from Wuhan Kanglu Co., Ltd. Procedure: Initially, tissue sections measuring 4 micrometers in thickness were subjected to an overnight heating process at 60°C to ensure proper fixation. Following this, the sections underwent a dewaxing process and were subsequently dehydrated through a series of graded alcohol solutions. The dehydrated sections were then immersed in distilled water and brought to a boil for thirty minutes to facilitate further preparation. Proteins were digested to a complete monomer at 37°C, followed by 5 minutes denatured at 83°C for hybridization. To create specific binding of the HER2 gene sequences, the denatured sections were then hybridized overnight at 42°C with the probe. Following hybridization, the sections were thoroughly washed to remove nonspecific binding and then dried ready to stain. Finally sections were re stained with DAPI, a fluorescent stain that associates to DNA to display nuclei. HER2 gene amplification was assessed by examining the prepared slides under a fluorescence microscope.

### 2.4 Statistical methods

Statistical analysis was done by SPSS version 22.0 and GraphPad version 9.0 software packages. In particular, the variability in HER2 expression was analyzed using GraphPad 9.0. Chi-square tests to determine the association of HER2 heterogeneity with clinicopathological features of patients were performed using both Pearson’s Chi-Square and Continuity Correction. Survival probabilities were analyzed using Kaplan-Meier estimator and significant differences across survival distributions were evaluated using log-rank test. Cox proportional hazards regression modelling was used to analyze prognostic variables with both univariate and multivariate analyses. Statistical significance was ascertained with a p-value below 0.05.

## 3 Result

### 3.1 HER2 expression in primary breast cancer and liver metastasis was heterogeneous

**Figure 1:**
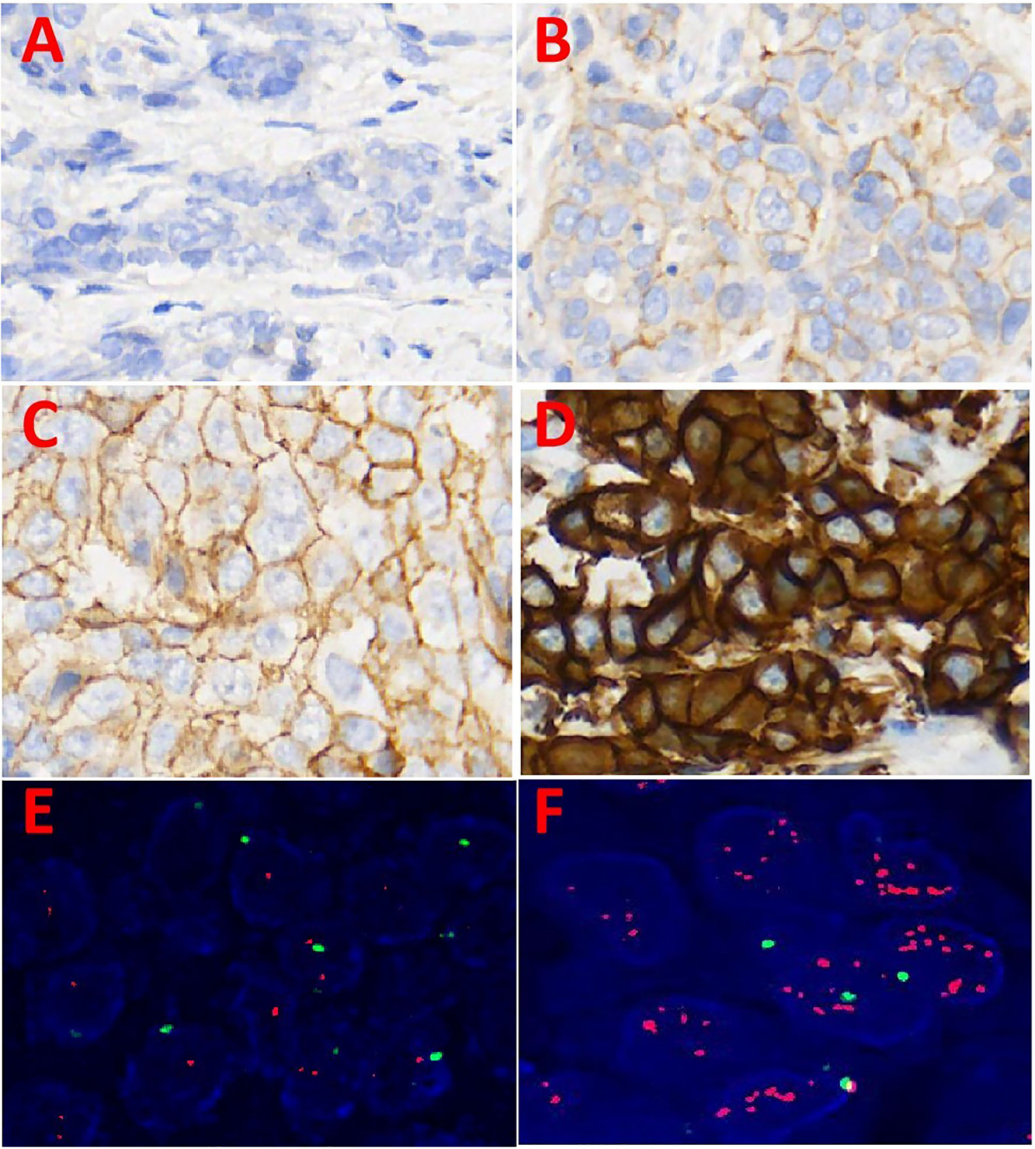
Immunohistochemical and fluorescence *in situ* hybridization analysis of HER2(200X). **(A)** HER2 immunohistochemical score 0: none or ≤10% of the cancer cells had incomplete, weak cell membrane staining; **(B)** HER2 immunohistochemical score 1+: > 10% of cancer cells showed incomplete, weak cell membrane staining; **(C)** HER2 immunohistochemical score 2+: > 10% of cancer cells showed weak to moderate strength intact cell membrane staining or ≤ 10% strong intact cell membrane staining; **(D)** HER2 immunohistochemical score 3+: > 10% of cancer cells showed strong, complete, uniform cell membrane staining; **(E)** HER2 FISH,No amplification of HER2(400X); **(F)** HER2 FISH, amplification of HER-2(400X).

**Figure 2:**
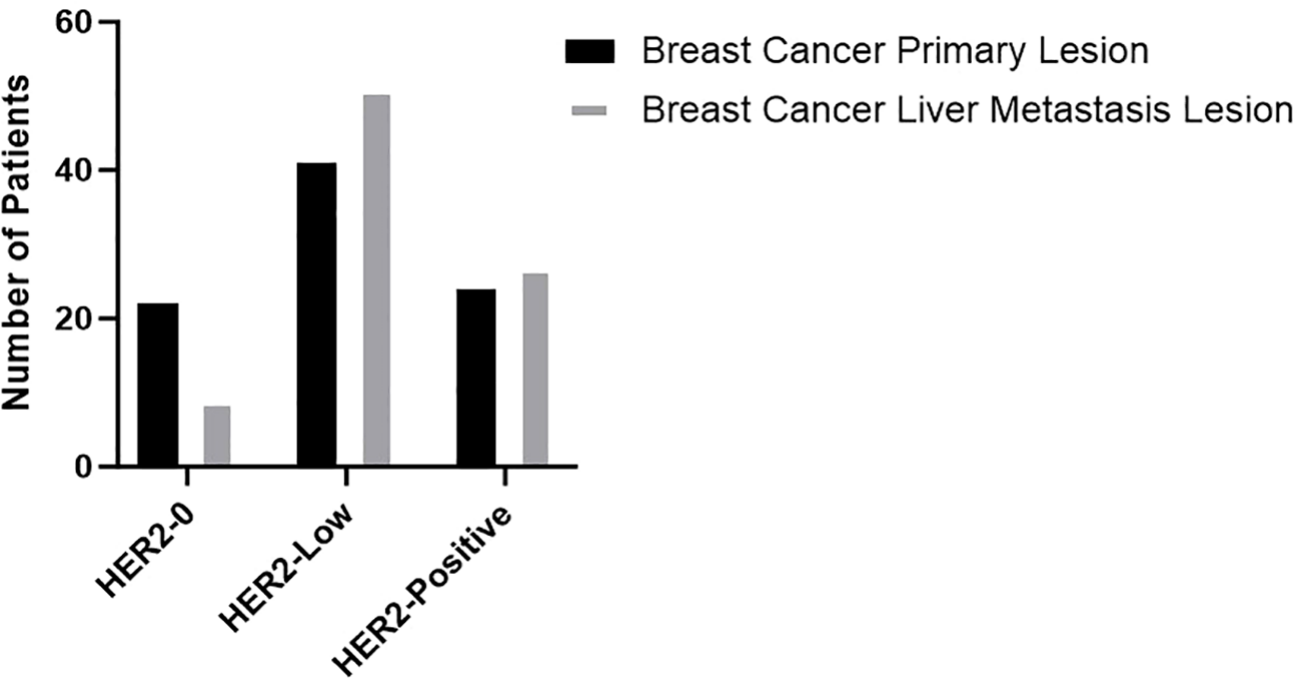
Number of patients of different HER2 expression in breast cancer primary lesion and breast cancer liver metastasis lesion.

HER2 expression in primary breast cancer and liver metastases was assessed using IHC and FISH techniques, with staining results shown in **Figure 1**. Among the 87 patients, 33 (38%) showed heterogeneous HER2 expression between primary breast tumors and liver metastases. This variation rate was significantly higher than the 20% reported by St Romain P et al., likely because their study only distinguished between HER2-negative and HER2-positive cases without including HER2 low expression (22). The distribution of HER2 expression patterns in primary breast cancer and liver metastases is shown in **Figure 2**. Among the 33 cases with heterogeneous HER2 expression, the most frequent change was from HER2–0 to HER2 low expression, observed in 17% of cases, as listed in **Table 1**. These findings highlight the importance of re-evaluating molecular markers in metastatic tumors. The availability of therapies that target HER2 low expression provides additional treatment opportunities for such these patients.

**Table 1:** HER2 heterogeneous expression between primary and liver metastases of breast cancer.

Group	HER2(n=87)					
	HER2 consistent	HER2 heterogeneity				
		0→L	0→H	L→0	L→H	H→L
Cases(n)	54(62%)	15(17%)	3(3%)	5(6%)	5(6%)	5(6%)

0→L represented HER2-IHC 0 expression in primary breast lesion transformed into HER2-low expression in liver metastases; 0→H represented HER2-IHC 0 expression in primary breast lesion transformed into HER2-positive expression in liver metastases; L→0 represented HER2-low expression in primary breast lesion transformed into HER2-IHC 0 expression in liver metastases; L→H represented HER2-low expression in primary breast lesion transformed into HER2-positive expression in liver metastases; H→L represented HER2-positive expression in primary breast lesion transformed into HER2-low expression in liver metastases

This study also analyzed the heterogeneous expression of ER and PR in primary breast tumors and liver metastases, with the results summarized in **Table 2**. Among the 87 patients, 14 (16%) exhibited ER heterogeneity, while 35 (40%) displayed PR heterogeneity. These rates were consistent with those variation rates reported by Reuben J. Broom and Mao Ding et al. (15, 23). Notably, the predominant trend for ER and PR heterogeneity was predominantly a shift from positive to negative expression. This pattern may result from the inherent heterogeneity of breast cancer cell populations and the selective expression of clonal receptors after treatment (24). Lara Schwieger et al. similarly suggested that the loss of ER/PR expression is common and may correlate with poorer survival outcomes (25). Routine evaluation of tumor markers in metastatic lesions is crucial for informing both prognostic assessments and therapeutic strategies.

**Table 2:** ER and PR heterogeneous expression between primary and liver metastases of breast cancer.

Marker	Primary	Liver	Cases (n)	HER2 Heterogeneity		P Value
				Yes	No	
ER Heterogeneity (n=14)	Negative→ Positive→	Positive Negative	3 11	2 7	1 4	>0.95
PR Heterogeneity (n=35)	Negative→ Positive→	Positive Negative	4 31	3 12	1 19	0.292

Primary represented breast cancer primary lesion; Liver represented breast cancer liver metastasis lesion; Negative→ represented Negative transfer to; Positive→ represented Positive transfer to

### 3.2 Relationship between HER2 heterogeneity and clinicopathological features of breast cancer patients

The association between HER2 heterogeneity and various clinicopathological characteristics of breast cancer patients, including histological type, histological grade, age, T stage, N stage, and overall survival (OS), was analyzed and summarized in **Table 3**. The results indicated that HER2 heterogeneity was correlated with patient age and ER heterogeneity. Specifically, patients aged ≥50 years and those with high ER heterogeneity were more likely to show HER2 heterogeneity.

**Table 3:** HER2 heterogeneity and clinicopathologic features.

Clinicopathologic feature		Cases(n)	HER2 heterogeneity		P Value
			Yes	No	
Histological type	IDC Other types	76(87%) 11(13%)	15(17%) 5(6%)	61(70%) 6(7%)	0.582a
Histological grading	I-II III-IV	50(57%) 37(43%)	7(8%) 6(7%)	43(49%) 31(36%)	0.380a
Age (y)	<50 ≥50	34(39%) 53(61%)	8(9%) 25(29%)	26(30%) 28(32%)	0.027a
T stage	T1-T2 T3-T4	80(92%) 7(8%)	31(36%) 2(2%)	49(56%) 5(6%)	0.900b
N stage	N0 N1-N3	58(68%) 29(32%)	18(21%) 15(17%)	40(46%) 14(14%)	0.061a
ER heterogeneity	Yes No	14(16%) 73(84%)	9(10%) 24(28%)	5(6%) 49(56%)	0.027a
PR heterogeneity	Yes No	35(40%) 52(60%)	15(17%) 18(21%)	20(23%) 34(39%)	0.437a
Survival time(y)	<5 ≥5	10(11%) 77(89%)	3(3%) 30(34%)	7(8%) 47(54%)	0.839b

IDC represented invasive ductal Carcinoma; Other types represented specific types of invasive ductal carcinoma;

a Pearson Chi-Square

b Continuity Correction

### 3.3 Analysis of correlation between HER2 heterogeneity and prognosis of breast cancer

**Figure 3:**
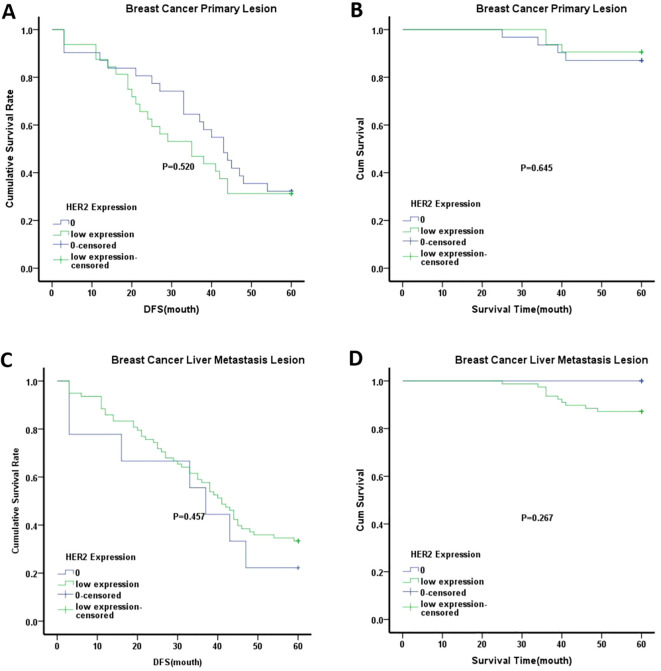
Kaplan-Meier analysis of different groups. **(A)** DFS of breast cancer primary lesion in HER2–0 group and HER2 low expression group; **(B)** OS of breast cancer primary lesion in HER2–0 group and HER2 low expression group; C.DFS of breast cancer liver metastasis lesion in HER2–0 group and HER2 low expression group; **(D)** OS of breast cancer liver metastasis lesion in HER2–0 group and HER2 low expression group. Note:0 represented HER2–0 expression group; low expression represented HER2-low expression group.

**Figure 4:**
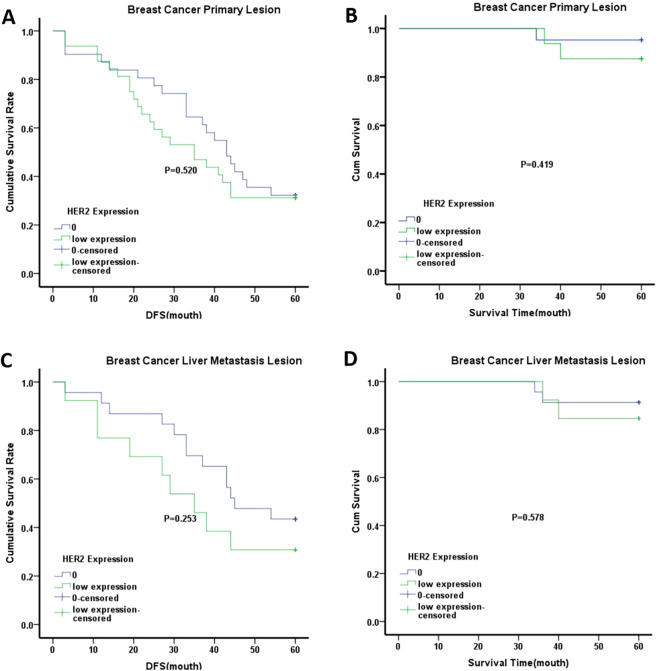
Kaplan-Meier analysis of different groups with ≥50 years old. **(A)** DFS of breast cancer primary lesion in HER2–0 group and HER2 low expression group; **(B)** OS of breast cancer primary lesion in HER2–0 group and HER2 low expression group; **(C)** DFS of breast cancer liver metastasis lesion in HER2–0 group and HER2 low expression group; **(D)** OS of breast cancer liver metastasis lesion in HER2–0 group and HER2 low expression group. Note:0 represented HER2–0 expression group; low expression represented HER2 low expression group.

Kaplan-Meier survival analysis was used to compare OS and DFS between the HER2-low expression and HER2–0 groups in primary breast tumors. The analysis showed no significant differences in OS or DFS between these groups, as shown in **Figure 3**. Similar results were observed in the liver metastases when comparing the HER2-low expression and HER2–0 groups. Interestingly, when stratified by age, the median DFS of patients aged ≥50 years in both primary breast tumors and liver metastases was lower in the HER2-low expression group than in the HER2–0 group. However, these differences were not statistically significant (**Figure 4**). Although the differences did not reach statistical significance, these findings suggest that HER2-low expression and HER2–0 expression may be associated distinct prognostic outcomes.

COX regression analysis was also conducted, and the results are presented in **Table 4**. The results indicated that PR heterogeneity emerged as an independent risk factor for DFS in both univariate and multivariate analyses. Patients with PR heterogeneity had poorer prognoses, with most changes in PR expression predominantly occurring from positive expression in primary breast tumors to negative expression in liver metastases. This observation supports the association between negative PR expression and poorer prognosis in breast cancer patients, aligning with the findings reported by Reiki Nishimura and colleagues (26).

**Table 4:** COX survival analysis.

Parameter	OS	P	DFS	P
	HR(95%CI)		HR(95%CI)	
COX univariate survival analysis				
Histological type	0.689 (0. 087-5.438)	0.724	1.289(0.553-3.005)	0.557
Histological grading	1.313 (0.380-4.537)	0.666	0.910(0.544-1.523)	0.720
Age (y)	1.006(0.284-3.566)	0.992	0.667(0.399-1.114)	0.122
T stage	0.766(0.097-6.044)	0.800	0.749(0.299-1.876)	0.537
N stage	4.532(0.574-35.773)	0.152	0.635(0.357-1.128)	0.121
ER heterogeneity	0.570(0.072-4.502)	0.594	1.353(0.642-2.851)	0.427
PR heterogeneity	0.418(0.089-1.966)	0.269	2.246(1.216-4.147)	0.017
HER2 heterogeneity	1.404(0.363-5.429)	0.623	1.438(0.838-2.466)	0.187
COX multifactor survival analysis				
PR heterogeneity			2.208(1.197-4.074)	0.011

P represented P value; OS represented Overall survival, DFS represented Disease-free survival.

## 4 Discussion

Breast cancer is the most prevalent malignancy worldwide and the leading cause of cancer-related mortality among women. Despite improvements in early detection and comprehensive treatment, 20%-30% of patients develop distant metastases, with a median survival of only 2 years for advanced cases (27–29). Specifically, breast cancer with liver metastasis carries a poor prognosis, with a median survival of 3–15 months and a 5-year survival rate of only 8.5% (30).

In addition to the challenges posed by distant metastases, the intrinsic heterogeneity of breast cancer during its progression greatly complicates treatment. This heterogeneity is manifested in several forms: the coexistence of different histological subtypes types within the same tumor, inconsistent expression of molecular markers between primary and metastatic lesions, and variations between biopsy results and the tumor’s complete molecular profile (13, 31).

Previous research has highlighted both the heterogeneity of breast cancer metastases and the organ-specific preferences of different molecular subtypes. Furthermore, metastatic tumors demonstrate unique organ-specific adaptations as they interact with the target organ’s microenvironment and proliferate. As a result, metastases in different organs may exhibit distinct molecular and phenotypic characteristics (32). Such heterogeneity may contribute to therapeutic resistance and disease recurrence. Therefore, identifying the biomarker profiles of recurrent or metastatic lesions is essential for guiding individualized treatment strategies in breast cancer.

A review summarized the results of 47 studies, and analyzed the heterogeneity in primary breast cancer and metastases of 3384 cases. They found that HER2 heterogeneity was about 10%. At the same time, they also found that the heterogeneity probability of different metastases was different, the bone metastases was the largest (33).Liu et al. also demonstrated that among 46 breast cancer patients with postoperative liver metastases, the inconsistency rate of HER2 expression in liver metastases compared to primary lesions was 10.9%. In contrast, our study found an inconsistency rate of 38% for HER2 expression in liver metastases. This discrepancy may be attributed to the inclusion of HER2 low expression in our evaluation, which was not considered in earlier studies. However, Ellen Yang et al. retrospectively analyzed the heterogeneous expression of HER2 in 147 breast cancer patients with diabetes from 2021 to 2022. The results showed that HER2 status was significantly inconsistent between the primary breast cancer and metastases,37% exhibited heterogeneous HER2 expression between primary breast tumors and metastases, most of which changed from HER2 low expression to HER2–0 expression (34).Federica Miglietta et al. analyzed the heterogeneous expression of primary breast cancer and metastases in 547 patients with breast cancer. They found that the total incidence of HER2 inconsistency was 38.0%, which was mainly manifested in the conversion of HER2–0 expression to HER2 low expression (15%) and HER2 low expression to HER2–0 expression (14%). HER2 low expression is highly unstable during disease progression (35).The HER2 heterogeneity rate in the studies of scholars such as Ellen Yang and Federica Miglietta is similar to our research results, both around 38%.Additionally, prior research has indicated that the heterogeneity of molecular marker expression varies, with HER2 considered relatively stable compared to the higher heterogeneity of PR. This difference may also stem from earlier studies focusing solely on HER2-negative and HER2-positive cases without accounting for HER2 low expression (36).

A recent large-scale study assessing the dynamics of HER2 low expression between primary and metastatic breast cancer reported that 28% of patients exhibited changes in HER2 status between the primary tumor and the first biopsy of recurrence or metastasis (37). In our study, HER2 heterogeneous expression between primary breast tumors and liver metastases reached 38%, suggesting that the inclusion of HER2 low expression in the analysis significantly increases the observed rate of HER2 heterogeneity. Such inconsistency in molecular marker expression not only affects breast cancer classification but also plays a critical role in determining the effectiveness of the initial treatment strategies.

Further correlation analysis between HER2 heterogeneity in liver metastases and clinicopathological features revealed an association between HER2 heterogeneity and patient age. This finding may reflect differences in the response and tolerance of older patients to treatment, potentially leading to more pronounced changes in HER2 status (38). Additionally, this study identified a positive correlation between HER2 heterogeneity and ER heterogeneity, indicating that HER2 heterogeneity may be influenced by hormone receptor status. Previous research has also shown that varying HER2 expression states under different hormone receptor conditions can result in distinct prognoses for breast cancer patients (39).

In this study, Kaplan-Meier survival analysis showed no statistically significant differences in OS or DFS between the HER2-low expression and the HER2–0 groups in either primary breast cancer or liver metastases. However, in patients aged ≥50 years, the median DFS was lower in the HER2-low expression group than in the HER2–0 group, both in primary and metastatic sites. Although these findings were not statistically significant, they suggest that patients with HER2-low expression and HER2–0 expression may be linked to different prognostic patterns, with age possibly serving as a contributing factor. Similarly, Mao Ding et al. similarly reported no significant difference in chemotherapy response between the HER2–0 and HER2-low expression subgroups (40). However, in a meta-analysis of 78,984 breast cancer cases across 17 studies summarized by Ciqiu Yang et al., it was reported that breast cancer patients with HER2-low expression had better survival outcomes than those with HER2–0 expression (41). Studies by Reiki Nishimura and colleagues reached similar conclusions (42). These controversies may be related to factors such as sample size and detection methods in different experiments, or due to limitations in the conditions at the time and the lack of the latest treatment methods (i.e. trastuzumab dulbactam). Subsequent COX regression analysis in this study identified PR heterogeneity as an independent risk factor affecting DFS. Patients with PR heterogeneity exhibited worse DFS, with PR expression frequently shifting from positive in primary breast tumors to negative in liver metastases. This finding indirectly supports the association of negative PR expression with poorer prognosis in breast cancer patients. This aligns with the findings of Li Zhuo et al., who reported a positive effect of high PR expression on breast cancer prognosis (43).

In addition to HER2 low expression, some researchers have introduced the concept of HER2 ultra-low expression, defined as HER2 (IHC between 0 and 1+) (11). Regardless of the expression level, accurate determination of HER2 status remains crucial and continues to present significant challenges for pathologists. In recent years, liquid biopsy techniques such as circulating tumor cells (CTCs) and free circulating tumor DNA (ctDNA), have emerged as non-invasive approaches for assessing HER2 status. These technologies offer the potential for safe, repeated evaluations and may complement conventional tissue biopsies in the future (44, 45). The 21-gene polygenic test has also been utilized to distinguish HER2-low tumors from HER2–0 tumors (46). Furthermore, imaging technologies, including [99mTc]Tc-hynicy-ly SPECT and ultrasonic radiographic features (URFs), have demonstrated the ability to assess HER2 expression in breast cancer patients (47, 48).

With the introduction of novel anti-tumor agents, breast cancer therapy has entered an era of precision stratification. Cyclin-dependent kinase 4 and 6 (CDK4/6) inhibitors have significantly improved the treatment of hormone receptor (HR)-positive breast cancer, while immune checkpoint inhibitors have reshaped therapeutic approaches for triple-negative breast cancer. Likewise, new anti-HER2 antibody-drug conjugates (ADCs) have achieved substantial success in HER2-positive breast cancer, profoundly changing clinical practice and treatment models (16–18). Ongoing efforts in the development of targeted therapies, identification of patient subgroups most likely to benefit, and strategies for addressing treatment resistance are progressively refining and optimizing breast cancer management.

This research is subject to several inherent limitations. Breast cancer typically has a favorable prognosis, which suggests that utilizing a five-year survival metric as the endpoint for follow-up might not adequately capture the long-term survival trends and outcomes. Prolonging the follow-up duration to ten years could potentially uncover more pronounced differences in survival rates and disease progression among the patient population. Moreover, the quantity of participants within the study significantly dictates the overall reliability and the broad applicability of its outcomes. Expanding the sample size is crucial as it would substantially reinforce the statistical rigor, thus enabling the derivation of more precise and conclusive insights from the data. Another important factor that could have affected the results is the inconsistency in IHC and FISH assay results, which may arise from variations in laboratory protocols, equipment, and technician expertise across different settings.

## 5 Conclusions

This study identified HER2 heterogeneity between primary breast cancer and liver metastases, with a rate reaching 38%. Although HER2 heterogeneity did not show a significant impact on prognosis of breast cancer patients in this cohort, accurate evaluation of molecular markers throughout disease progression remains essential. Future research should place greater focus on assessing HER2 status in distant metastases to improve the precision and effectiveness of treatment strategies. In addition, ongoing developments in liquid biopsy technology present a promising approach for monitoring HER2 status and guiding individualized therapy. As our understanding of the mechanisms underlying HER2-targeted therapy mechanisms advances and new agents continue to emerge, these progressions will bring new opportunities and hope for patients with breast cancer.

## Data Availability

The original contributions presented in the study are included in the article/supplementary material. Further inquiries can be directed to the corresponding authors.
